# Ovine Herpesvirus 2 Infection in Foal, Brazil

**DOI:** 10.3201/eid1505.081664

**Published:** 2009-05

**Authors:** Érica A. Costa, Maria Rosa Q. Bomfim, Flávio G. da Fonseca, Betânia A. Drumond, Fabiana Magalhães Coelho, Anilton C. Vasconcelos, Ronaldo Furtini, Tatiane A. Paixão, Renee M. Tsolis, Renato L. Santos, Mauricio Resende

**Affiliations:** Universidade Federal de Minas Gerais, Belo Horizonte, Brazil (É.A. Costa, M.R.Q. Bomfim, F.G. da Fonseca, B.A. Drumond, F.M. Coelho, A.C. Vasconcelos, T.A. Paixão, R.L. Santos, M. Resende); Instituto Mineiro de Agropecuária, Belo Horizonte (R. Furtini); University of California, Davis, California, USA (R.M. Tsolis)

**Keywords:** Viruses, malignant catarrhal fever, horses, goat, Brazil, letter

**To the Editor:** Malignant catarrhal fever (MCF) is an acute, generalized, and usually fatal disease previously thought to be restricted to mammals of the order Artiodactyla, often members of the subfamilies Bovinae, Cervidae, and Suidae (*1*). Although animals of the order Perissodactyla, family Equidae, have previously been considered not susceptible to ovine herpesvirus 2 (OvHV-2), we show that horses may be infected by this virus.

In July 2006, in the state of Minas Gerais, Brazil, neurologic signs developed acutely in a 6-month-old foal; signs included muscle spasms, rigidity of the neck and limbs, difficulty standing, and hind-limb paralysis. The foal also had severe dyspnea and profuse sweating and died 1 day after the onset of clinical signs.

Histopathologic findings included a marked lympho-histiocytic fibrinoid necrotizing vasculitis affecting small blood vessels and arterioles in the kidney and liver (Figure) and associated with lymphocytic interstitial nephritis and mild multifocal granulomatous hepatitis. The vasculitis lesions were strikingly similar to those observed in cattle with MCF caused by OvHV-2 or alcelaphine herpesvirus 1. Both forms of the disease have a wide spectrum of clinical manifestations, but histopathologic findings for the 2 forms are similar (*2*,*3*). In addition, the foal had severe and diffuse interstitial pneumonia characterized by thickening of the alveolar walls and interstitial accumulation of macrophages, proliferation of type II pneumocytes, and accumulation of cell debri in the alveolar lumen. Granulomatous inflammation, characterized by mild multifocal to coalescent accumulation of epithelioid macrophages, was also observed in the spleen and lymph nodes. Surprisingly, the brain showed only moderate congestion and mild, multifocal, perivascular hemorrhage.

**Figure:**
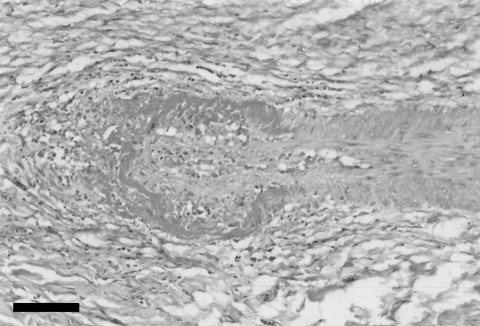
Liver of foal with vasculitis associated with intralesional ovine herpesvirus 2 DNA, showing segmental, severe, fibrinoid, necrotizing arteriolitis. Hematoxylin and eosin stain. Scale bar = 100 μm.

Although horses are not considered susceptible to OvHV-2, histopathologic findings in this case were consistent with MCF-like lesions. Thus, we looked for any history of direct or indirect contact between the affected foal and goats or sheep. Indeed, on this particular farm, horses shared food with 65 goats. On the basis of indirect contact with potential reservoirs of infection, the disease in the foal was suspected of being associated with a member of the MCF virus (MCFV) group (*4*).

To determine whether the disease in the foal was associated with an MCFV, we obtained tissue samples from the foal at necropsy and collected blood from all 3 adult horses, including the dam, and 10 randomly selected goats on the farm. DNA was extracted from these samples, and PCR was performed to detect members of the MCFV group, including OvHV-2 (*5*), caprine herpesvirus 2 (*6*), and alcelaphine herpesvirus 1 (*7*). The tissues from the foal as well as peripheral blood mononuclear cells from all 3 adult horses and 8 tested goats were positive for OvHV-2 only. The adult horses had no clinical signs of infection for at least 8 months after the outbreak.

To confirm the PCR detection of OvHV-2, we purified amplicons obtained from the dam of the affected foal, the affected foal, and 1 goat and processed them for automated sequencing. These nucleotide and deduced amino acid sequences were identical, and we deposited them in GenBank under accession nos. EU244694, EU718486, EU718487. PCR was conducted on tissue samples of the foal to test for differential diagnosis agents equine herpesvirus 1 (*8*), equine herpesvirus 4 (*8*), and equine arteritis virus (*9*). No amplification was observed. Considering that sheep and goats are the most important natural reservoirs of OvHV-2 (*6*), these results support the notion that infected goats were the most likely source of infection for horses in this outbreak.

To further support this diagnosis of OvHV-2 infection, we attempted to detect viral DNA in the vascular wall of the foal’s hepatic arterioles that contained fibrinoid necrotizing lympho-histiocytic vasculitis by using laser capture microdissection on sections stained with hematoxylin and eosin (*10*). DNA extracted from these areas was PCR positive for OvHV-2, which confirmed the co-localization of OvHV-2 DNA sequences in the site of MCF-like lesions.

Taken together, these findings confirm an emergent infectious disease associated with OvHV-2 infection in a horse, a species previously considered not susceptible to OvHV-2. The finding of vasculitis associated with intralesional OvHV-2 DNA sequences unequivocally demonstrates the pathogenic potential of this virus in foals. However, a cause-and-effect relationship between OvHV-2 infection and interstitial pneumonia as well as the granulomatous inflammation in the liver and spleen could not be established in this case. This report supports the notion that either equine infection is extremely rare or that this strain of OvHV-2 underwent recent modifications that expanded the host range.
